# New Ways of Approaching Indoor Residual Spraying for Malaria

**DOI:** 10.9745/GHSP-D-16-00354

**Published:** 2016-12-23

**Authors:** Michael Macdonald

**Affiliations:** a WHO Emergency Response to Artemisinin Resistance Hub, Phnom Penh, Cambodia.

## Abstract

Using health extension workers in Ethiopia as supervisors of the spray team reduced operational costs while maintaining quality. But rethinking IRS calls for (1) adapting equipment and procedures to ensure higher-quality spray applications, and (2) empowering decentralized targeting against malaria transmission foci.

See related article by Johns.

The article by Johns et. al. on indoor residual spraying (IRS) for malaria control in Ethiopia, published in this issue of GHSP,^1^ presents a variation on standard IRS operating procedures by essentially replacing the “squad leader,” usually recruited from the district town, with the health extension worker (HEW) from the community. The squad leader supervises the spray operators, who are also recruited from their own communities rather than from various parts of the district as in standard operations. In this new community-based IRS model, other supervisory structures above the level of the squad leader—from the district, zonal, and regional offices—were kept in place. The stated goal of moving from district-based to community-based IRS implementation was cost savings, and indeed there were marginal savings to the operational costs associated with this change. In this era of new, more costly insecticides developed to manage insecticide resistance while programs simultaneously shift from broad implementation of malaria “control” to more targeted malaria “elimination,” the work by Johns and colleagues raises several important issues.

## COSTS

Indeed, the average cost per person protected in the community-based IRS districts was lower than in the district-based model—US$0.87 vs. $1.00, respectively, in 2013 and $0.86 vs. $1.03 in 2014. Moreover, there was a shift in costs from transportation expenses (with the money presumably going to a vehicle rental company) to the daily wages of workers (which benefited the local economy). It appears these figures may just be for the cost of the spray campaign itself and not the overall cost of the program. From a separate report, the same author indicated the overall cost per person protected in Ethiopia was $5.33, of which the spray campaign itself comprised 18.7% of the total cost while the cost of the bendiocarb insecticide was 52.1% of the overall cost.^2^ As programs shift to the newer “next generation” insecticides, unit costs for insecticide could increase over and above the cost of bendiocarb, and certainly over the cost of earlier insecticides including DDT and pyrethroids. While the Ethiopian community-based IRS model showed incremental cost savings and it was also good to shift input to local wages, there are two other issues this strategy bring up.

Cost of the insecticide comprises the largest portion of the overall costs of a spray campaign.

## QUALITY

Quality of the spray application is the Achilles' heel of IRS operations. Most programs use compression sprayers first developed in the 1940s; some still use a “stirrup sprayer” developed even decades earlier. Furthermore, our multimillion dollar IRS operations still remain entirely dependent on the diligence of the spray operators, temporary workers often paid less than $5/day, to apply the right dose to the right surface. Concern over quality led to much skepticism on the use of HEWs as squad leaders, but the results reported in this issue of GHSP suggest that HEWs are able to deliver a quality spray operation comparable with their district-level counterparts.

Quality of the spray application is the Achilles' heel of IRS operations.

Still, we need to develop application equipment less prone to “operator error.” In the more than 60 years since the compression sprayer was developed, there has been a revolution in spray application technology in agriculture, automotive painting, ink-jet printers, and facility disinfection. Some of these include incremental improvements to the existing compression sprayers with Constant Flow Valves (now used by programs supported by the U.S. President's Malaria Initiative), to radically different technologies such as electrostatic spray nozzles that produce charged spray droplets to get up and under to stick to the target surface. Investments in malaria diagnosis, treatment, and prevention must also focus on disruptive technologies for this oldest mainstay of malaria control, the hand compression sprayer.

## FLEXIBILITY AND TARGETING FOR MALARIA ELIMINATION

The third issue touched on in the Ethiopian IRS project was flexibility and the ability of HEWs, as members of the community, to “use their local knowledge of the demarcations of malaria-affected and malaria-free parts of villages to target spray areas more effectively than in the DB [district-based] model.”

Like the compression sprayer itself, the structure and functions of IRS operations come from the 1950s post-war environment where many of the malariologists were former members of the military medical corps, and so adopted much of the language and logistical structures of a military campaign: centrally planned “geographical reconnaissance,” “attack phase, consolidation and maintenance phase,” “squad leaders,” etc. And like large military campaigns, most IRS operations follow a rigid timetable of operations set in motion months in advance.

Shifting from “control” to “elimination” requires a quantum leap forward in our use of epidemiological, entomological, and environmental mapping to target interventions and eliminate foci. On the national scale, we consider epidemiological and entomological surveillance systems, GIS, and remote sensing technologies for risk-area stratification. In the Ethiopian communities using the community-based IRS model, it was the knowledge and experience of the HEW that provided the flexibility and ability to target spray operations. Now that a pocket-size mobile phone has as much computing power as a desktop from a decade ago, we need to link the two—the mobile phone with the community worker.

Shifting from malaria control to elimination requires a quantum leap forward in our use of technology to target spray interventions and eliminate foci.

## RETHINKING IRS

One must recognize that Ethiopia's Health Extension Program is exceptionally strong, and establishing such community-based structures may be a challenge in other malaria-endemic areas. One must also be cognizant that HEW supervision of IRS implementation may have an opportunity cost—taking time away from their other essential duties. And finally, of course, “pilot projects” always run the risk of not being sustainable when taken to a larger scale. Nevertheless, the project shows that in addition to the incremental cost savings from the community-based model, there could be some new thinking in the way we have approached IRS for the past 6 decades.

Recognizing that quality of the spray application is the critical element of our multimillion dollar investments, we need to develop or adapt spray technologies from other sectors to enable decentralized, minimally trained and supervised operators to deliver a correct dose of insecticide to the appropriate surface. The same is true for *Aedes* control where “standard” IRS is of limited efficacy; dengue, chikungunya, and Zika control programs are exploring new ways for “targeted IRS” for the particular indoor harborages of *Aedes*, such as closets and behind furniture. New application equipment that can deliver a more quality-assured spray would enable programs more flexibility and confidence that much of the operations can be decentralized to more community-based structures, including the HEWs in Ethiopia.

## Notes

**First Published Online:** 2016 Dec 13

## References

[1] Johns B Yihdego YY Kolyada L Dengela D Chibsa S Dissanayake G Indoor residual spraying delivery models to prevent malaria: comparison of community- and district-based approaches in Ethiopia. Glob Health Sci Pract. 2016;4(4):529–541. 10.9745/GHSP-D-16-00165 27965266 PMC5199172

[2] Johns B Cico A PMI IRS country programs: 2014 comparative cost analysis. Bethesda (MD): Abt Associates, Africa Indoor Residual Spraying Project; 2015 Available from: https://www.pmi.gov/docs/default-source/default-document-library/implementing-partner-reports/africa-indoor-residual-spraying-project-pmi-irs-country-programs-2014-comparative-cost-analysis.pdf?sfvrsn=4

